# Modeling Dynamic Regulatory Processes in Stroke

**DOI:** 10.1371/journal.pcbi.1002722

**Published:** 2012-10-11

**Authors:** Jason E. McDermott, Kenneth Jarman, Ronald Taylor, Mary Lancaster, Harish Shankaran, Keri B. Vartanian, Susan L. Stevens, Mary P. Stenzel-Poore, Antonio Sanfilippo

**Affiliations:** 1 Pacific Northwest National Laboratory, Richland, Washington, United States of America; 2 Oregon Health & Science University, Portland, Oregon, United States of America; Institute for Systems Biology, United States of America

## Abstract

The ability to examine the behavior of biological systems *in silico* has the potential to greatly accelerate the pace of discovery in diseases, such as stroke, where *in vivo* analysis is time intensive and costly. In this paper we describe an approach for *in silico* examination of responses of the blood transcriptome to neuroprotective agents and subsequent stroke through the development of dynamic models of the regulatory processes observed in the experimental gene expression data. First, we identified functional gene clusters from these data. Next, we derived ordinary differential equations (ODEs) from the data relating these functional clusters to each other in terms of their regulatory influence on one another. Dynamic models were developed by coupling these ODEs into a model that simulates the expression of regulated functional clusters. By changing the magnitude of gene expression in the initial input state it was possible to assess the behavior of the networks through time under varying conditions since the dynamic model only requires an initial starting state, and does not require measurement of regulatory influences at each time point in order to make accurate predictions. We discuss the implications of our models on neuroprotection in stroke, explore the limitations of the approach, and report that an optimized dynamic model can provide accurate predictions of overall system behavior under several different neuroprotective paradigms.

## Author Summary

Computational modeling aims to use mathematical and algorithmic principles to link components of biological systems to predict system behavior. In the past such models have described a small set of carefully studied molecular interactions (proteins in signal transduction pathways) or larger abstract components (cell types or functional processes in the immune system). In this study we use data from global transcriptional analysis of the processes of neuroprotection in a mouse model of stroke to generate functional modules, groups of genes that coherently act to accomplish functions. We then derive equations relating the expression of these modules to one another, treating these individual equations as a closed system, and demonstrate that the model can be used to simulate the gene expression of the system over time. Our work is novel in describing the use of global transcriptomic data to develop dynamic models of expression in an animal model. We believe that the models developed will aid in understanding the complex dynamics of neuroprotection and provide ways to predict outcomes in terms of neuroprotection or injury. This approach will be broadly applicable to other problems and provides an approach to building dynamic models from the bottom up.

## Introduction

The ability to examine the behavior of biological systems *in silico* through time and under different conditions has the potential to greatly accelerate the pace of scientific discovery in biology. Wet lab experimental work on disease pathologies such as stroke in animal model systems is both time intensive and costly. The ability to develop computer models based on high-throughput measurements of the system that can be interactively perturbed to test system behavior under diverse simulated conditions would greatly reduce the time and cost of experimental work by identifying hypotheses that are most likely to lead to promising lines of inquiry. For example, substantial effort has been recently devoted to understanding the system biology of neuroprotection in stroke by studying the transcriptomic responses prior to and following cerebral ischemia and the alterations induced by the application of neuroprotective preconditioning stimuli [1], [2], [3]. This work has yielded extensive gene expression data on the genomics of neuroprotection in diverse contexts and can be used to train dynamic pathway models of neuroprotection in stroke. Such dynamic models can in turn be used to simulate additional experimental conditions by manipulating variables such as removing or changing the expression of regulatory influences in order to investigate corresponding alterations in the molecular processes of neuroprotection over time. The ability to carry out such simulations can help identify hypotheses about the underlying mechanisms of neuroprotection that may have been unrealized or considerably reduce the time and effort that would have been needed to reach the same conclusions through *in vivo* and *in vitro* experiments.

Within the last decade, there has been a slow but steady growth in the application of dynamic modeling to represent biological systems including metabolic networks, regulatory networks, and signal transduction pathways. Mandel et al. (2004) provides an exemplification and discussion of a host of candidate techniques for modeling dynamic biological processes with reference to an idealized representation of the lac operon [4]. These techniques include; ordinary differential equations (ODEs), Petri nets, Boolean networks, dynamic Bayesian networks, signal-flow diagrams, agent-based modeling, and system dynamics. Of these techniques, dynamic Bayesian networks [5], [6] and Boolean Networks [7], [8] have been more widely used, but Petri nets [9] and agent based modeling have also started to make an impact [10]. Many of these approaches have been applied to small, well-characterized systems, such as the lac operon, but not to model global data from transcriptomics. Modeling approaches that involve ODEs have traditionally focused on expert knowledge to supply parameters for small networks of genes, but methods have been developed to infer regulatory networks from high-throughput transcriptomic data [11], [12], [13], [14], [15].

Our approach introduces several novel elements to the dynamic modeling research described above. First, we derive our model structure and parameters from high-throughput global transcriptional data using a network inference method coupled with an optimization process. Second, we work with a set of regulatory relationships that are representative of an entire system. Finally, the computational model describes stroke and neuroprotection in a higher eukaryote, the mouse. Our resulting ODE-based model has several advantages over other modeling approaches including the inclusion of feedback loops, which are important for many biological processes, and the ability to predict the temporal patterns of gene expression. Our goal in adopting this approach is to model changes in expression levels of functional modules over time, and to assess the interactions between these functional modules as regulatory influences, in order to facilitate interactive simulations of neuroprotection during cerebral ischemia.

**Figure 1:**
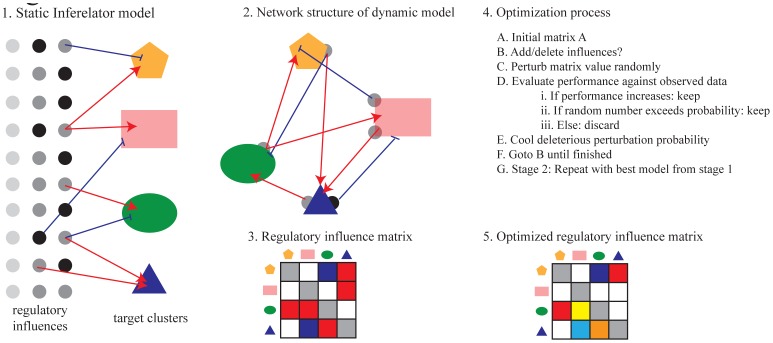
Overview of abstract dynamic modeling approach. 1) Inferelator 1.1 infers a parsimonious set of potential regulatory influences whose expression can explain the expression of the target cluster maximally, but does so independently for each cluster. 2) The actual structure of the inferred network would consider that the regulators are members of clusters and that the network structure is complex and cyclical. 3) The regulatory influence model can be represented by a regulatory influence matrix and used to simulate the closed system of ordinary differential equations over time. 4) The optimization process (see text) is used to improve the ability of the model to simulate the system over time (i.e. calibrate to temporal data). 5) The resulting optimized model retains much of the structure of the initial model.

In this paper we describe an approach to generate dynamic systems models of networks of functional modules using predicted causal influences from temporal transcriptomics data (Figure 1). We first identified co-expressed gene clusters that represent functionally coherent modules. The structure and initial parameters of the model (in the form of ODEs) were generated using a method that learns steady-state relationships between regulators and their targets based on expression data, the original Inferelator algorithm [14], [16]. A simulated annealing method was applied to the system of ODEs that represents the initial model, to optimize parameters for dynamic simulation over time. We found that the optimized models perform quite well at simulating the patterns of expression that they were trained on, providing a proof of concept that the closed systems of ODEs is capable of accurately representing expression in a complex system over long time scales. Validation of the models on time course data not used for optimization produces limited, but significant performance results showing that the model is accurately capturing aspects of general regulation in the system. Additionally, we validated the relationships in our model by examining correspondence with interactions from other sources. We show that this process can produce high-level functional models from high-throughput data that are capable of accurately describing the dynamics of the transcriptomic response in blood to neuroprotective stimuli and to cerebral ischemia over time.

## Results

### Model data

Gene expression data obtained from microarrays (Affymetrix) run on mouse blood was utilized for this study (see Methods). The mice were treated with one of three different preconditioning stimuli: lipopolysaccharide (LPS), CpG-oligonucleotide (ODN), or brief ischemia (15 minute stroke); or treated with saline or a sham surgical procedure as controls. These preconditioning stimuli provide the brain with a defined window of protection against cerebral ischemia. The protection requires time to develop prior to the ischemic event to allow for the necessary new gene expression and protein synthesis to develop. In this model, the preconditioning stimuli or control treatments were given three days prior to stroke, which was induced surgically using transient middle cerebral artery occlusion (MCAO). Following stroke, preconditioning leads to a neuroprotective effect demonstrated by decreased infarct (ischemic injury lesion) size [1], [17], [18]. It has been shown that preconditioning dramatically affects gene expression in the brain [1] and blood ([19] and unpublished observation) prior to and following stroke. To understand the dynamics of the blood changes in gene expression, microarrays were run on blood samples collected at 3, 24, and 72 hours following preconditioning and at 3 and 24 hours following stroke. Though this is a limited number of time points to parameterize regulatory models, the dataset provides a good starting point for establishment of methods for predictive modeling in complex eukaryotic systems. Particularly, the five parallel time courses with different pretreatment conditions are valuable to assess the ability of resulting models to predict novel behavior.

### Defining functional modules using expression patterns across multiple conditions

**Figure 2:**
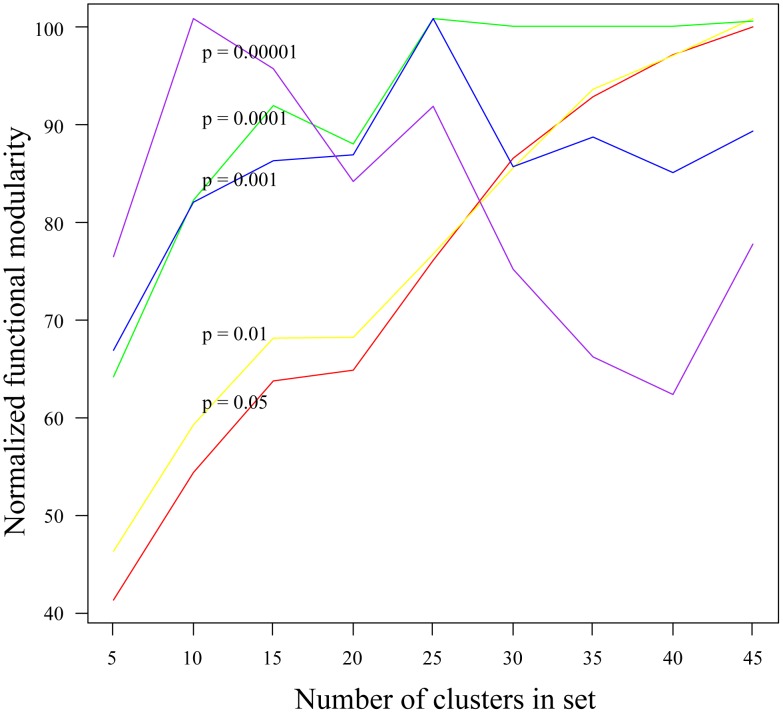
Functional coherence of clusters defines functional modules. To ascertain a reasonable number of clusters to consider in our model abstraction we calculated the normalized functional modularity (Y axis) for varying numbers of clusters (X axis) from the same hiearachical tree derived from the expression data. Functional modularity was defined as the number of genes annotated with a biological process gene ontology category that was functionally enriched in the gene's parent cluster with a p-value less than the threshold indicated (colored lines). The results show that 25 clusters provides a peak of functional modularity, especially for more coherent functional categories with lower p-values.

To establish the actors in the model with predictable expression we first defined functional modules from the set of significantly changing probes (see Methods). Functional modules are clusters of co-expressed genes that have coherent functions. To define these functional modules we clustered the expression data from all treatments considered using a hierarchical clustering approach. This process produces a dendrogram that can be used to divide the data into an arbitrary number of clusters. To determine the clustering division (i.e. the number of clusters) that best represents functional modules in the system we assessed the functional coherence of sets of clusters at different clustering divisions. Specifically, for any given clustering we determined the functional enrichment of each cluster in terms of biological process as described in the Gene Ontology (GO) [20]. All categories that were found to be significantly enriched (using several p-value thresholds, see Figure 2) in a cluster were considered to be enriched categories. The functional coherence was then calculated as the total number of genes that were annotated with at least one enriched category. This approach provides a simple, yet intuitive measure of how coherent functional groups are in the clustering structure defined from the data. The results of this analysis show that maximum functional coherence is achieved with 25 clusters (Figure 2). We found that the mean in-cluster correlation in expression between genes for all 25 clusters was 0.59 whereas the mean between-cluster correlation was 0.28, showing that the expression patterns were also largely coherent in these clusters. It is important to define functional modules because the utility of the model depends on the predicted outputs (mean expression levels of functional modules defined here) reflecting coherent biological functions that are executed by the system in response to the treatment. Though many of the clusters represented are too large to be considered true functionally coherent modules, we felt that this was a good starting point for further model development given the coherence of the functional categories and expression patterns for this set of conditions.

### Initial steady-state model inference

To learn the parameters in a model system of ODEs that relate the expression levels of clusters to one another we applied a modification of the original Inferelator [14], [16] algorithm (version 1.1). This algorithm uses an approach called L1 error regression (also known as Lasso) to choose a parsimonious set of regulatory influences that can explain the expression of each cluster maximally [21], [22]. We have previously described elements of this approach in this and other systems [23], [24], [25], [26]. The result is a model abstracted from high-throughput transcriptomic data that mathematically expresses the relationships between clusters. To enable dynamic simulation models, our approach infers the influence of clusters on each other but excludes individual regulator genes (such as transcription factors) as explicit regulatory influences. The original Inferelator 1.1 approach includes individual regulator genes, but is not designed to enable dynamic simulation. It is capable of limited temporal prediction when based on time course data, but does not consider relationships between different ODEs in the model. The Inferelator approach was extended to enable inference for such dynamic, coupled systems and Bayesian parameter estimation was applied. The resulting models were capable of predicting the expression profiles in yeast over time with good accuracy [13]. Similarly, our abstraction (depicted in Figure 1) allows the model to be treated as a closed, coupled system of ODEs where the expression levels of all clusters at any given time step can be calculated from their expression levels at the initial time. The resulting model can be simulated over time given only an initial starting state (see below), which is not possible using the original Inferelator approach.

The initial steady-state model can be used to predict the expression of target clusters given the known expression levels of the input regulatory influences (in this case the regulatory influences are other clusters). Although the Inferelator is capable of incorporating time course data explicitly in its inference process, we chose to treat data from each time point as a steady-state measurement since the time steps were relatively long and of variable length. We assessed the performance of the model using a cross-validation approach in which multiple models are trained on subsets of the data, by leaving out the data corresponding to a treatment time course, and then evaluated on the excluded time course data as previously described [23], [24], [25], [26]. Prediction of the expression levels for a cluster in this steady-state model requires input of expression levels for the regulatory influences, in this case the clusters themselves, for each condition being predicted. Using this approach we found that there was relatively high correlation of 0.73 (range of −1.0 to 1.0) between the predicted and observed expression levels, across the 25 conditions examined (five preconditioning treatments at five time points each). Considering only the LPS preconditioning time course, the correlation was somewhat higher at 0.78, as were the other time courses alone: CPG-ODN (0.80), ischemic preconditioning (0.82), saline treatment (0.92), and sham surgery (0.81). We have previously reported similar results using models developed with the Inferelator that consider individual genes as regulatory influences, rather than entire clusters [23], [26]. These models can be used to perform limited types of simulations, such as predicting expression of target clusters after *in silico* deletion of a regulatory influence. However, a significant limitation of this kind of model is that the expression levels of the input regulatory influences must be measured for every time point in order to be able to make predictions. Our dynamic model only requires specification of the initial state to simulate the subsequent time steps, potentially for a broad range of different initial conditions.

### Dynamic simulation of expression levels using an inferred model

We were interested in determining if the initial steady-state model generated by the Inferelator could be used as the basis for dynamic simulation to describe how the expression levels of all clusters in the model change over time. Accordingly we transformed the model into a matrix of coefficients in which the rows refer to regulatory influences and the columns refer to their targets. This matrix is the basis for a linear system of ODEs that can be solved in closed form for specific parameter choices, and more generally using standard ODE solvers. The resulting dynamic model was used to simulate the expression levels of the target clusters in the system given only one known input variable, which was the initialization state from the first time point in each preconditioning treatment. The simulated expression profile was then compared to the observed expression profile for that preconditioning treatment for each cluster at multiple times using correlation as a basis of comparison as above. We used a correlation measure for optimization, as opposed to a more standard measure such as the root mean square deviation (RMSD), because we are more interested in capturing the pattern of expression rather than the magnitude of the fold-change in expression. We found that this dynamic model using only the Inferelator-derived structure and parameters yielded a correlation with the observed data of only 0.36 for LPS and similarly poorly for the other time courses (Table 1). The results are not surprising given that the inference approach employed by Inferelator 1.1 considers clusters independent from one another, and not as a system of coupled ODEs.

**Table 1:** Optimization results.

Pretreatment	Inferelator^a^	Dynamic^b^	Optimized^c^	BestCross^d^
**LPS**	0.78	0.36	0.71	0.48
**CpG**	0.80	0.26	0.80	0.56
**Preconditioning**	0.82	−0.32	0.67	0.24
**Saline**	0.92	0.66	0.79	0.51
**Sham**	0.81	−0.34	0.71	0.10

a Performance of the Inferelator-based model in steady-state prediction.

b Performance of the Inferelator-based model in dynamic prediction.

c Performance of the best optimized model for that pretreatment.

d Performance of the best model optimized to another pretreatment.

**Figure 3:**
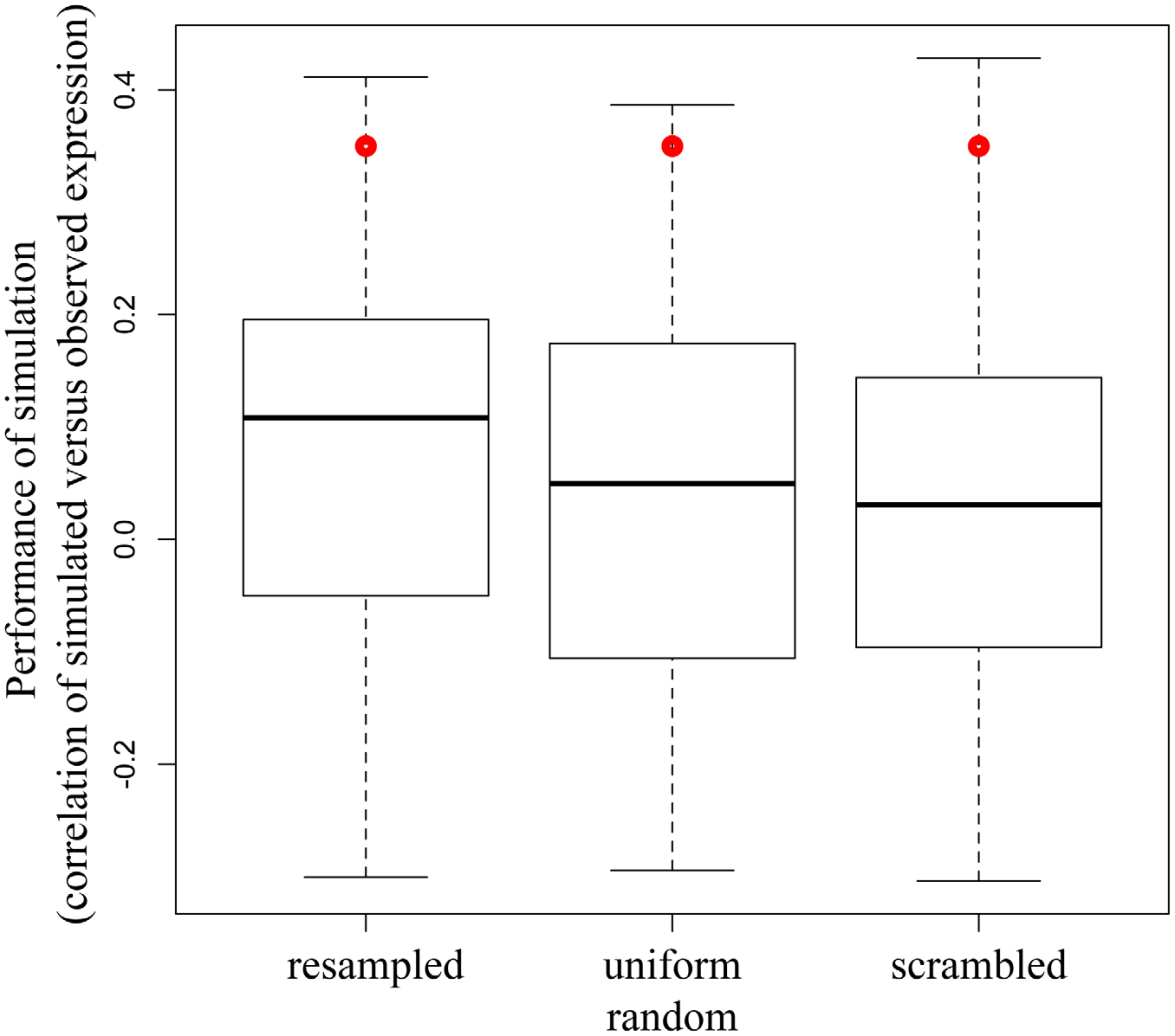
An Inferelator-based influence model provides statistically significant performance when treated as a system of ordinary differential equations (ODEs). The Inferelator-based influence model was treated as a system of ODEs and simulated over time. Expression levels of the simulation were compared with observed values of expression by correlation (Y axis) for the initial model (red dot) or for 100 randomized matrices. The matrices were randomized by replacing all non-zero values with other non-zero values (resampled) or from a uniform distribution (uniform), or the locations of all values in the matrix were reassigned (scramble). The results (as a box and whiskers plot) show that the Inferelator-based initial matrix produces simulation over time with a performance that is significantly better than that using random permutations of the matrix.

Though the performance of the dynamic model using parameters derived by Inferelator 1.1 was moderate, we were interested in determining if the model contained useful information, in the form of its coefficients and/or structure that could be used as a basis for further dynamic model development. Two aspects of the model are critical in determining how accurately it simulated temporal profiles: its structure in terms of the pattern of regulatory influences between clusters, and the coefficients of each of these regulatory influences that form the system of ODEs. We investigated the information content of the coefficients of the model as well as the structure of the model by performing several different randomizations of the existing model. We first preserved the structure of the model but perturbed the coefficients for each edge (nonzero values) by randomly resampling all nonzero values from the initial matrix. We calculated the mean (0.087) and standard deviation (0.167) of the correlation measures for 100 such resampled matrices and found that the performance of the initial matrix (0.36) was significantly different from the performance of the model using the randomized matrices (p-value<0.05), indicating that the model contains significant predictive value. To examine this result further we resampled coefficients in the model from a uniform distribution for nonzero values, which produced similar results. We next investigated the structure of the network by randomly permuting all values in the matrix. This process creates a new structure of regulatory influences between the clusters, but preserves the overall number of such influences between clusters as well as the distribution of coefficients. The mean (0.029) and standard deviation (0.149) of the correlation measures using 100 such scrambled matrices indicate that the model using the initial matrix had significantly different performance than models using scrambled matrices (p-value<0.02), suggesting that the initial model derived using the ODEs from Inferelator contains significant value in the form of its structure as well. These results are summarized in Figure 3.

### Optimization of model for dynamic simulation

To improve the performance of the dynamic model we employed simulated annealing to optimize the initial matrix against observed patterns of expression from the data. Similar regulatory network model parameter estimation was accomplished using a Metropolis-Hasting Monte Carlo approach in a Bayesian formulation [13]. Simulated annealing randomly perturbs model parameters (the regulator/cluster coefficients in this case), and then compares the performance of the perturbed model with the original using a fitness function. Perturbations that improve performance are retained in the model and deleterious perturbations can be retained to help ensure greater exploration of parameter space. Retention of some short-term deleterious perturbations is based on a probability that is decreased over the simulation, resulting in gradually more conservative changes to the model as annealing proceeds. Our fitness function evaluates the performance of each test matrix by the correlation of its simulated expression values with the observed expression values. We used each of the different pretreatment time courses for the optimization, initializing the model with expression levels at the 3 hour time point, as described above. Optimization was carried out in two rounds, using the best model from the first round as the starting point for the second round, as described in Methods. Addition and removal of regulatory influences was allowed to explore alternate structures for the model. As in [13] we also optimized α parameter for each cluster that represents a cluster expression degradation rate, to account for factors like normal mRNA degradation and turnover. The second round begins with a lower probability of accepting deleterious perturbations to allow some exploration of parameter space without large deviation from the initial matrix. An optimized model should provide much better performance than the initial model, and may refine the structure of the initial model to accomplish this. We found that when the probabilities for addition and removal were both set to a value of 0.01 the resulting models were much more complicated (i.e. they had more components connected). Comparing the performance of the model resulting from optimization using these probabilities with one generated when the edge addition probability was set to 0.001 revealed that more conservative edge addition resulted in a more parsimonious model with higher performance. Hence, we used this value in the current study.

**Figure 4:**
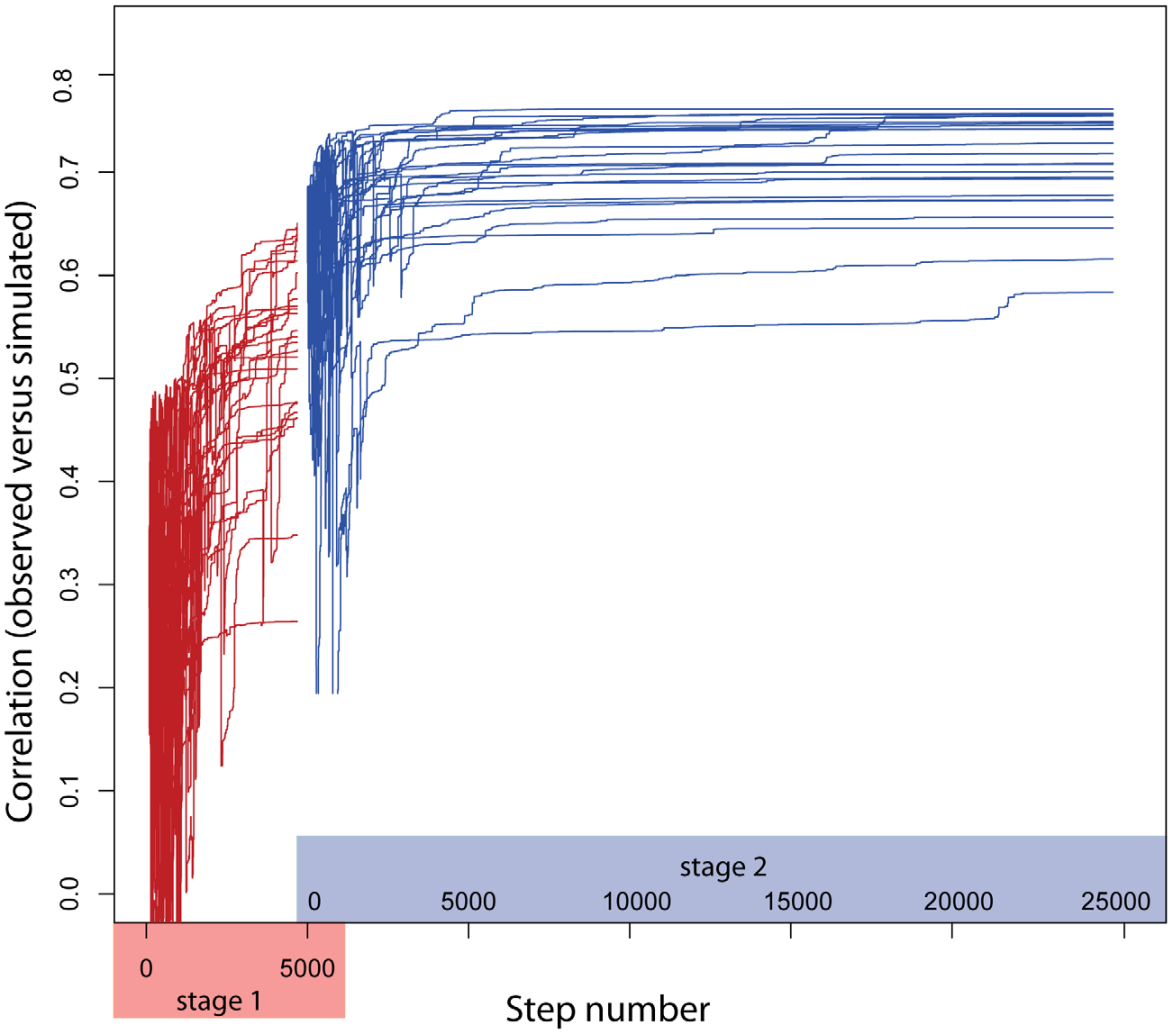
Performance trajectories of models during simulated annealing process. The two-stage simulated annealing (SA) process described was applied using the Inferelator-based model as a starting matrix. The performance (Y axis) of each of the 25 models in each stage are shown over the steps (X axis) in the SA process. The results show that the optimization process can dramatically improve the performance of the initial model.

We optimized the initial matrix against each of the time courses from individual pretreatments separately, and show the results from the LPS-optimized matrix here (all results are presented in Table S1). The results of the optimization process for the LPS time course are shown in Figure 4. This plot shows the evolving performance of each optimization run, for both rounds of the process. The best dynamic model resulting from this process has a performance (correlation with observed expression profile) of 0.71 which is comparable to performance given by the steady-state Inferelator-based model, when evaluated against the LPS treatment time course that was used in the optimization procedure. It is important to stress that the steady-state Inferelator model requires the expression levels of input regulatory influences to make predictions of the expression levels of the target clusters and, in this case, the regulatory influences are other clusters, which is very different from the dynamic model as it requires only an initial state. This demonstrates that it is possible to develop a dynamic model that can accurately simulate the expression levels of all clusters over time with very good accuracy from an initial steady-state model inferred from transcriptional data.

**Figure 5:**
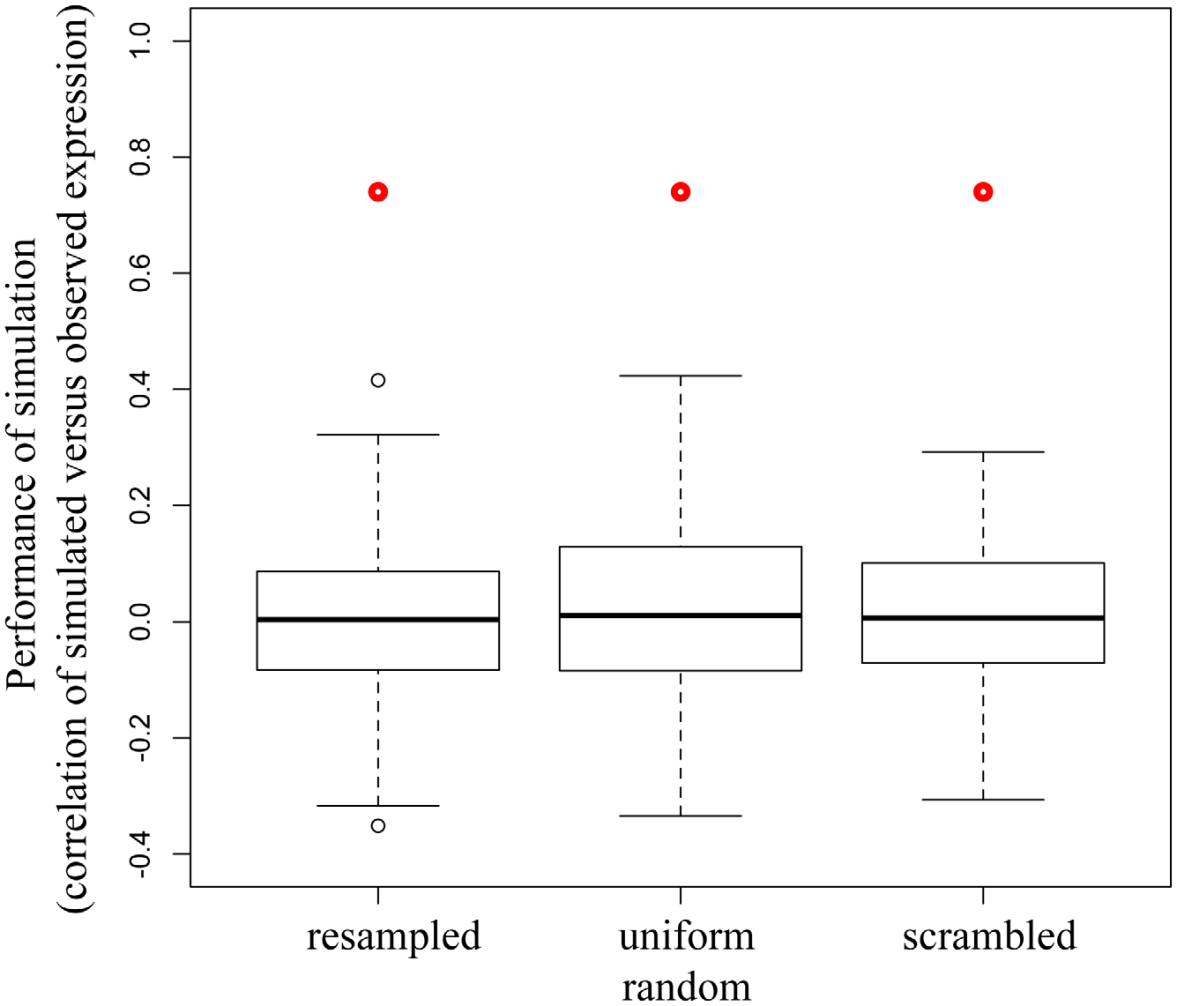
The optimized model performs significantly better than randomly perturbed models. The best optimized model was simulated over time to provide predictions of expression levels for clusters. Correlation (Y axis) of the simulated versus observed data is shown for the best optimized model (red dot) and for 100 randomized matrices (boxes). The matrices were randomized by replacing all non-zero values with other non-zero values (resampled) or from a uniform random distribution (uniform), or the locations of all values in the matrix were reassigned (scramble). The results (as a box and whiskers plot) show that the optimized model is capable of simulation over time with a performance that is significantly better than randomized versions.

We examined both the significance of the performance obtained in the optimized model and the sensitivity of the optimization result to the initial matrix for model optimization to each pretreatment. For the former we randomized the optimized model as described above for the initial model. Results indicate how significant the performance of the optimized model is relative to models with randomized coefficients and randomized structures. We first examined the performance of the best model relative to scrambled and randomized versions of the same matrix (as above), resampling coefficients but preserving model structure (resampled and uniform random in Figure 5) and restructuring the model (scrambled). These results for the LPS pretreatment are shown in Figure 5 and show that the optimized model has a significant predictive value over all the randomized models (p-value<1e-7).

**Figure 6:**
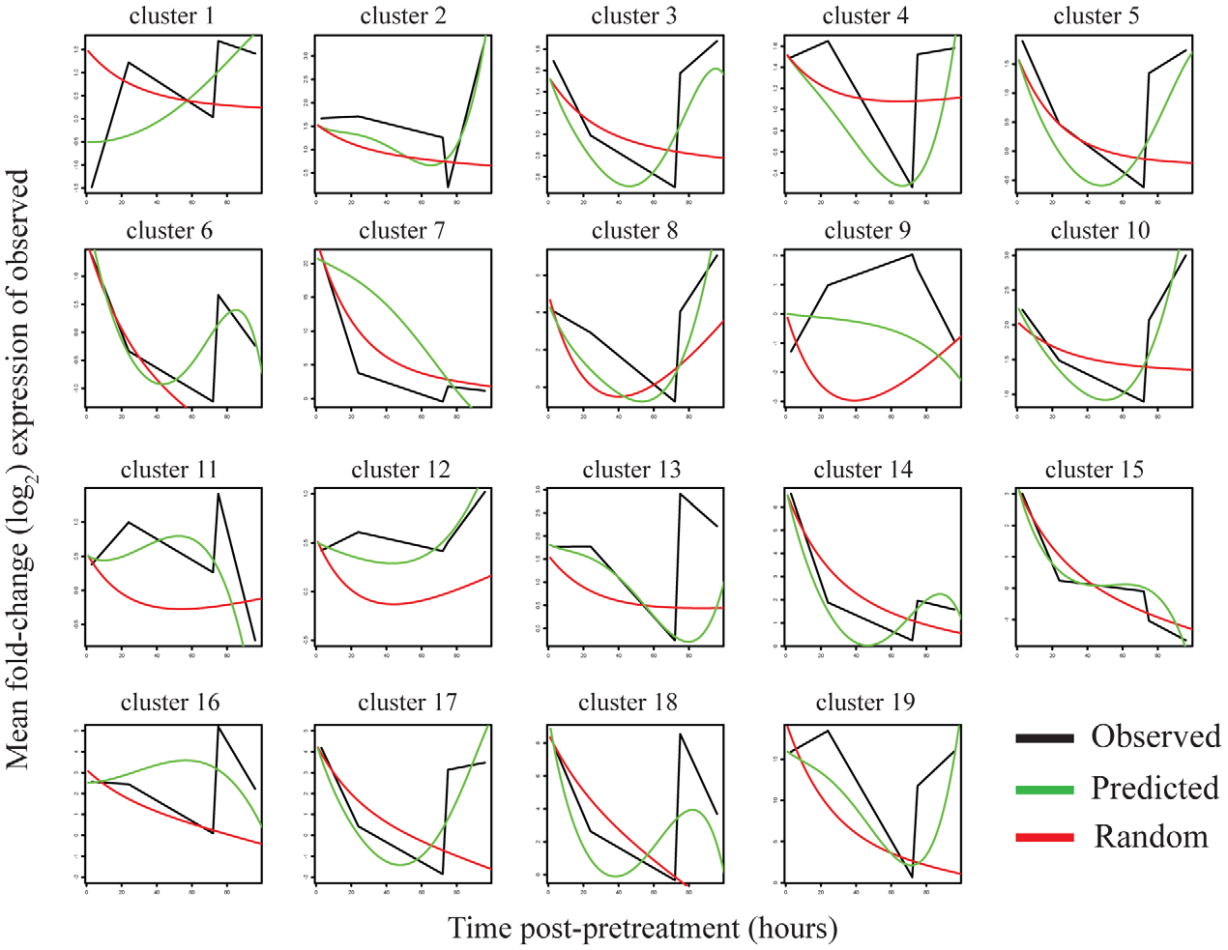
Expression patterns for an LPS-optimized model. The LPS-pretreatment observed (black lines) and predicted (green lines) expression patterns for clusters containing more than five genes are shown. The expression patterns from a randomized consensus model (red lines) are also shown. The X axes indicate the log2 fold-change expression for the observed pattern and the predicted and random expression patterns were scaled to this range. The Y axis shows time from 0 to 100 hours post-pretreatment.

The expression patterns predicted by the LPS-optimized model are shown in Figure 6. For each cluster with more than five genes we show the observed (black line), predicted (green line), and random (red line) expression patterns for the LPS time course. For the random prediction we show the average prediction from 25 randomly restructured models (coefficients randomized in the model). These results show that the model can do quite well at recreating many of the patterns of expression of the time course on which it was optimized.

Our approach generates an ensemble of 25 models. We wanted to compare these models and determine if a combined model might give better performance than individual models. Accordingly, we assessed the variability in the model structures in the ensembles by counting the numbers of times each edge is represented in the ensemble. We found that there is a large degree of concordance between the models in terms of their structure, but that some edges seem to be present in all models whereas other edges are represented in only a subset of models. The distribution of edge counts is shown in Figure S1 and the consistency of the edges in the model is shown in Figure S2. The absence of edges with low counts (1–15) in part reflects the structural decision we made to limit the probability of adding edges in the optimization process. Additionally we found that the range of weights for each edge in the ensemble was quite large (Table S2). This is likely due to the fact that the models are optimized using a fitness measure based on correlation, and this means that the magnitudes of predicted expression values (and thus model edge weights) can vary

To examine the performance of combined models we took edge weights of the combined model as either the mean or median of weights from the ensemble. Either of these approaches resulted in models with performance that was representative of the performances in the ensemble for the time course used in the optimization, but not better than the best model in the ensemble. We examined performance of these models both on the time course used for optimization as well as the other time courses (cross-validation) with similar results (see Table S1).

To test for sensitivity to the initial matrix, we optimized using randomly selected initial models. This was to determine how important the initial model provided by the Inferelator is to the final optimized performance. We repeated the optimization process starting with 25 matrices with the same structure as the original matrix, but with the weights randomized. After the two rounds of optimization we found that the mean performance of the model using the 25 final matrices was slightly lower than those produced by optimization using the original initial matrix (mean 0.60 versus 0.66, respectively, p-value 2e-7 by t-test) indicating that the initial weights are important to the final outcome of the optimization. This shows that the optimization works well even when starting with a randomized model, but performs significantly better when the initial model is provided by the Inferelator.

We next examined the question of whether the structure of the initial model was important to the outcome of the optimization process. We randomized the structures 25 times by randomly permuting the initial weights in the matrix and proceeded with the optimization process. After the second round of optimization we found that the performance of the 25 final matrices was significantly worse than those produced by optimization of the original matrix (0.62 versus 0.66, p-value 5e-3) indicating that the initial structure provided by the Inferelator is very important to the outcome of the process. Though the optimization process itself allows restructuring of the model, it is unlikely that large-scale restructuring will take place since individual changes (addition or removal of an edge) are evaluated individually. Thus modifications to the initial structure of the model are expected to be conservative (see Conclusions).

### Evaluation of optimized model

Thus far, to evaluate the optimized dynamic model, we applied it to the same data that had been used for the optimization process to determine whether it was successfully predictive. However, this can result in overstatement of results due to overfitting data. To more rigorously evaluate the performance of the dynamic model we determined the performance of the best model optimized against the LPS preconditioning time course on the dataset for each of the other conditions. The results of this analysis indicate that the model can provide reasonable predictions of behavior in the LPS (correlation 0.74), CpG-ODN (0.41, p-value 0.01) and saline (0.39, p-value 0.004) time courses but that it fails to accurately predict behavior under the brief ischemia preconditioning treatment (correlation 0.01) and sham treatment. To assess the similarity between responses in each treatment we calculated the correlation between gene expression ratios from all differentially expressed genes at each comparable time point from different treatments, and report the results as the mean correlation between treatments (Table 2). This shows that the time courses are somewhat correlated with each other, but retain enough differences to provide a good evaluation of the models' abilities to generalize. The performance of each cluster for each time course examined is shown in Table 3.

**Table 2:** Correlation of gene expression between pretreatment time courses.

	LPS	CpG	Pre	Saline	Sham
LPS	—	0.617	−0.039	0.394	0.700
CpG	0.617	—	0.113	0.592	0.754
Preconditioning	−0.039	0.113	—	0.063	0.049
Saline	0.394	0.592	0.063	—	0.689
Sham	0.700	0.754	0.049	0.689	—
**Mean**	**0.418**	**0.519**	**0.047**	**0.434**	**0.548**

**Table 3:** Performance of LPS-optimized model on individual clusters.

			Simulation Performance				
Cluster	Functional label	Gene Count	LPS	CpG	IP*	Sham	Saline
cluster_1	apoptosis	587	0.57	0.49	−0.49	0.50	−0.43
cluster_2	hemopoiesis	275	0.93	0.71	0.09	−0.49	−0.47
cluster_3	cell migration/blood coagulation	1324	0.80	−0.76	−0.38	−0.42	0.63
cluster_4	cell division/defense response	934	0.61	0.22	−0.41	0.79	−0.80
cluster_5	metabolic process	824	0.79	0.15	0.85	−0.57	0.39
cluster_6	cell differentiation	280	0.77	−0.33	0.70	−0.17	−0.61
cluster_7	inflammatory response	58	0.75	0.81	0.87	−0.59	0.34
cluster_8	cell differentiation	84	0.82	0.64	−0.47	0.90	−0.39
cluster_9	NK cell/leukocyte mediated immunity	140	0.08	−0.16	0.19	0.82	0.08
cluster_10	blood coagulation	410	0.88	0.91	0.32	0.80	−0.55
cluster_11	inflammatory response	637	0.79	0.75	0.54	−0.09	0.43
cluster_12	mitosis	510	0.88	0.74	0.08	0.70	−0.54
cluster_13	innate immune response	474	0.10	0.27	−0.54	0.47	−0.35
cluster_14	inflammatory response	129	0.97	0.86	0.73	−0.56	0.50
cluster_15	apoptosis	288	0.97	0.99	0.64	0.50	−0.32
cluster_16	cell differentiation	84	0.04	0.09	0.43	−0.48	0.40
cluster_17	development	226	0.75	−0.18	0.96	0.67	0.47
cluster_18	response to stimulus	52	0.57	0.43	0.51	−0.45	0.40
cluster_19	immune response	22	0.79	0.81	−0.52	0.75	−0.45
cluster_20	-	4	0.32	0.61	−0.44	0.86	−0.08
cluster_21	-	5	0.97	0.65	0.47	−0.08	0.53
cluster_22	-	1	0.53	−0.25	0.47	−0.75	0.30
cluster_23	-	1	0.95	0.38	0.59	−0.42	0.53
cluster_24	-	1	1.00	0.74	0.02	0.63	−0.19
cluster_25	-	1	0.45	0.68	−0.37	0.93	−0.24

* IP, ischemic preconditioning.

**Figure 7:**
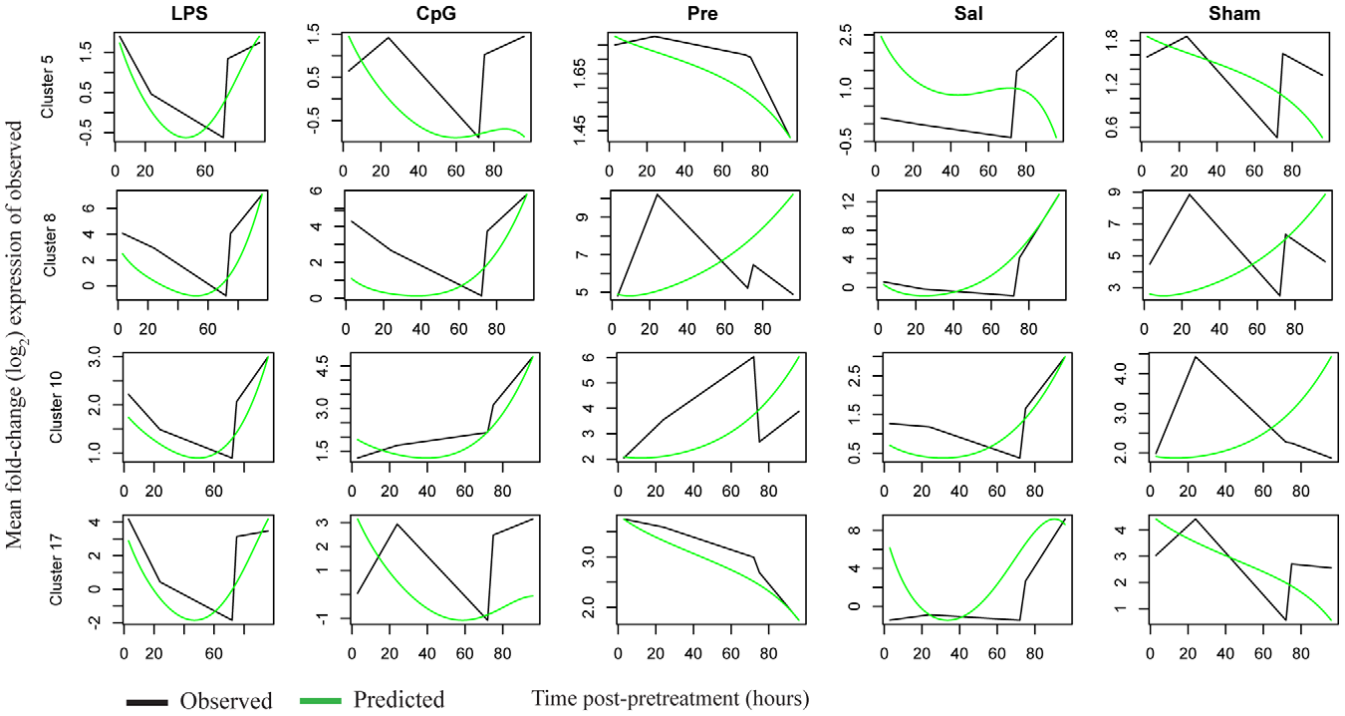
Cross-predictive performance of an LPS-optimized model. Relative expression levels (log_2_ fold change expression) are plotted over time (X axis) for the predicted (green line) and observed (black line) expression levels for the indicated cluster. Several representative clusters are shown and the remaining plots are included as Figure S3.

The simulated and observed expression values from the LPS-optimized model are shown in Figure 7 over the different preconditioning treatment time courses for several representative clusters. Predictions for the remaining clusters are presented as Figure S3. While several predicted patterns for other time courses are consistent with the data, many other predicted patterns are not. Nevertheless, this result shows that for some clusters the optimized model can approximately capture patterns of gene expression under multiple preconditioning treatments, even when those patterns are quite different. The correlation values for these plots are shown in Table 3. It is clear that simulations for some of the clusters do not change from treatment to treatment (clusters 8 and 10 in Figure 6) but others display markedly different patterns of expression under the different pretreatment conditions (clusters 5 and 17 in Figure 6).

### Validation of inferred model edges using orthogonal interaction data

Although our inferred and optimized model seems to be consistent with existing data, at least within the limitations of the results presented above, we were concerned that this could be due to overfitting of the data and/or to our model being one of many possible consistent models that is not necessarily biologically relevant. Our derived model does not make specific predictions of gene-to-gene interactions or influences, but rather relates the general expression patterns of clusters of genes. Therefore to examine the consistency of this model with data from external sources we used the following strategy. We used interaction data from four independent data sources; regulatory binding site interaction from chromatin immunoprecipitation (ChIP) experiments [27], physical protein-protein interactions [28], known gene regulatory network neighborhoods [29], and high-confidence inferred functional relationships from integrated data [30]. For each cluster in our model we determined the number of known interactions between a gene/gene product in the cluster and genes in each of the other clusters. To determine a p-value for the interactions we counted interactions gathered by randomizing the known edges for each external interaction dataset 1000 times. Those cluster-to-cluster relationships with a p-value of less than 0.05 were considered to be true positive (TP) matches if there was a corresponding edge in our inferred model for each interaction dataset, and true negatives (TN) if the p-value was greater than 0.05 and there was no inferred edge. The accuracy for each dataset was calculated as TP+TN/(TP+TN+FP+FN) and these results are presented in Table 4. We present the complete results from this analysis for each cluster-to-cluster relationship as Table S2.

**Table 4:** Interaction datasets validate network model.

Dataset	Accuracy
CHIP	60.9%
PPI	66.8%
Regulatory	64.3%
Functional	64.3%
Any	82.0%

These results show that each independent dataset has a modest correspondence with our inferred model ranging from 61–67%. Each of these different sources of interaction data is limited, either by the coverage it provides or by the amount of accuracy it might have. Therefore we evaluated the maximum accuracy obtainable by combining results from each interaction dataset by simply counting a match as a true positive or true negative if it was validated by any interaction dataset. While this method would not be appropriate to evaluate the prediction accuracy since it would be impossible to choose *a priori* which interaction dataset result to choose, it does provide a reasonable way to validate our model based on its overlap with existing interaction data. The combined accuracy of 82% shows that while the model is not perfectly aligned with these existing datasets, it seems to be fairly consistent with existing biological knowledge.

We also found that the number of protein-protein interactions linking genes inside a cluster were highly significant, with most clusters having a p-value of 0 (out of the 1000 random counts; see Table S2). This is in contrast to the ChIP-based regulatory interactions, which mostly did not have interactions within clusters. These observations support our initial validation of functional modules (Figure 2) and indicate that the clusters in our model are coherent functional modules that are regulated coordinately and exert regulatory influences on other functional modules.

### Comparison of injurious and non-injurious predicted models

**Figure 8:**
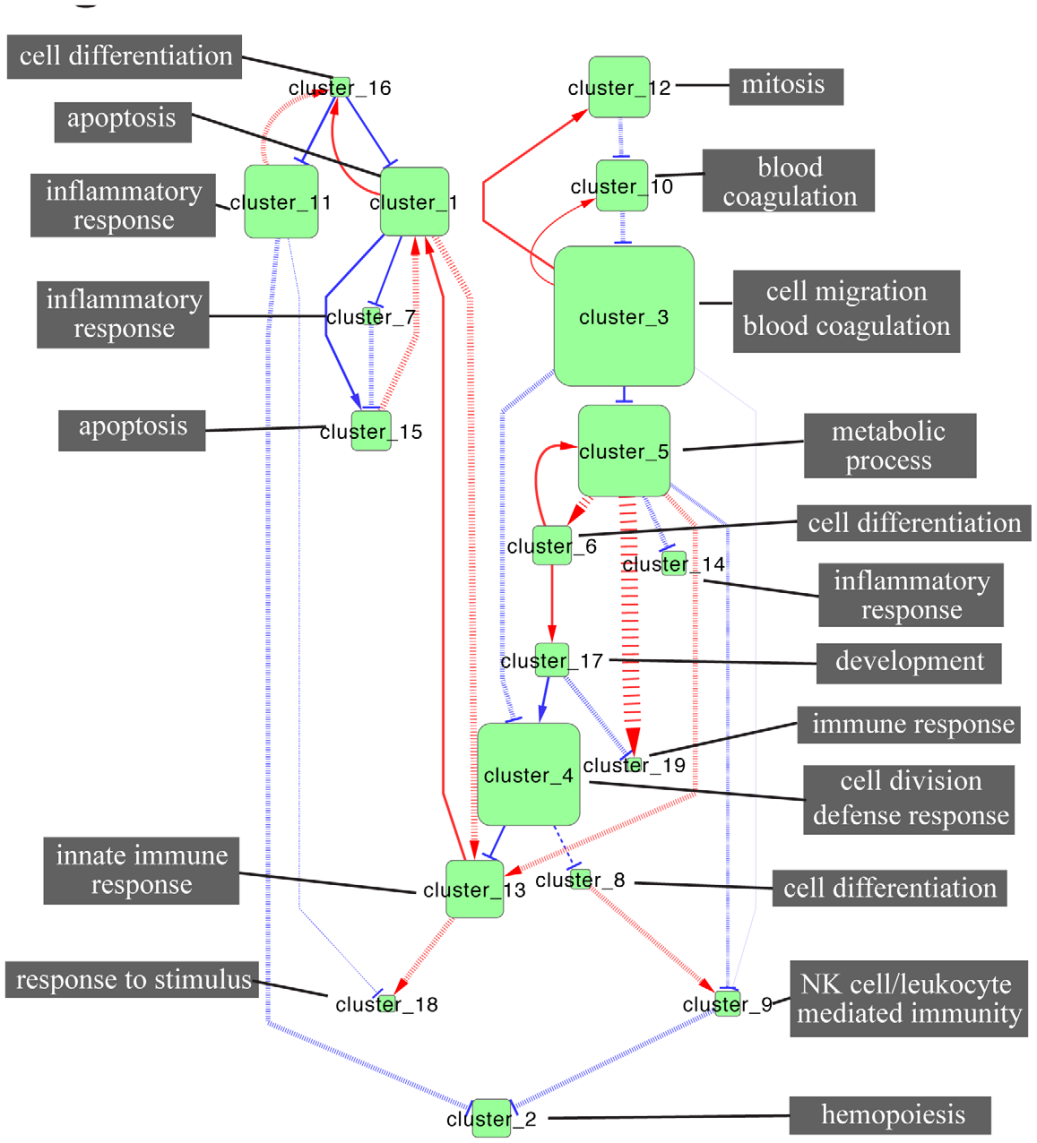
Comparison between neuroprotected and injurious networks. Clusters containing more than 5 genes are shown (green squares) as determined by our functional clustering approach. The influences between clusters are shown as directed edges with red arrows indicating a positive influence (activation) and blue T lines indicating a negative influence (repression). Dashed lines indicate relationships that are significantly different between injurious and non-injurious conditions, either absent in one or opposite sign. General functional categories chosen from statistically enriched functions are indicated in grey boxes.

A key question in neuroprotection and stroke concerns the regulatory differences between injurious conditions (no pretreatment) and non-injurious (neuroprotective pretreatment). By grouping together like conditions we can gain insight into these differences. We did this by assessing the concordance of models generated for each set of injurious conditions (saline and sham treatment) and models generated for each set of non-injurious conditions (LPS, CpG or brief ischemic pretreatment). An edge was considered to be present in the final injurious or non-injurious model if it was present in 50% or greater of the models from each of the member conditions from either group (that is, one or two models in the injurious set and two or three in the non-injurious set). The weight of the final edge was taken as the mean of weights from the sets, with the primary goal of assessing differences in differences in the weights between injurious and non-injurious models, either in terms of presence/absence of an edge or a reversal in function from activation to repression or vice-versa. A comparison of the resulting models is shown in Figure 8. In this figure solid lines represent model edges that are conserved between both conditions in terms of presence and sign, dashed lines represent edges that are present in one but not the other condition (see Table S3 for details), and cross-hatched lines indicate edges where the sign is opposite in each condition. This analysis highlights that the outgoing edges from cluster 5 are all different between injurious and non-injurious conditions. This suggests that genes or subsets of genes in cluster 5 may play an important role in induction of neuroprotection during stroke. These results are discussed further below.

## Discussion

In this paper we demonstrate how a steady-state model derived from high-throughput data that describes inferred relationships between regulatory influences and their targets can be used to produce dynamic simulation models capable of making accurate predictions over extended periods of time. We explored the ability of these models to predict expression under conditions not used for optimization and found the predictive ability, while limited, to be significant. This work represents an advancement in the application of dynamic models that are based on high-throughput transcriptional data sampled at low temporal resolution. The model depends on considering groups of coexpressed and functionally related genes as both the targets of regulatory influences and as the originators of these influences. This has the advantage of creating a simple closed model for which the transcriptional levels of all regulatory influences are predicted and allows the model to function as a system of ODEs that can be solved using standard tools. The steady-state model produced by the original Inferelator requires input of observed expression levels of the regulatory influences to make predictions, limiting its utility for making novel predictions. That is, at a given time or in a given condition, the regulators must be measured in order for the gene expression values of the functional modules to be predicted by the steady-state model. Although our approach requires using the observed data for initial model inference, the resulting dynamic model is capable of simulating other treatment time courses given only the initial state of the system, or extrapolating to further time points not measured. Additionally, the dynamic model couples ODEs used in the initial steady-state model, allowing explicit temporal evolution of the system. Calibrating the dynamic model to observed expression levels produces results that are shown to be significant, indicating that it can provide a good starting place for further optimization to produce a better model.

Using our optimized dynamic model, we found that the relationships between regulatory influences and their putative target clusters can be used to simulate the system over time with statistically significant performance. Our modeling approach represents a way to derive dynamic models from steady-state static models that represent an abstraction of the system from high-throughput transcriptional data. Previous efforts similar to this have focused on very detailed models (for example [31]) or very high-level abstract models (for example [32]) that incorporate a large amount of preexisting knowledge to establish relationships between components, generally not incorporating high-throughput data. A notable exception is work by Madar, et al. describing an enhancement of the original Inferelator algorithm that is capable of dynamic simulations similar to our approach [13]. These approaches provide an alternative to previous methods by allowing the creation of models capable of simulating the expression levels of important functional processes over time derived in a largely automated fashion from high-throughput data. The resulting models can then serve as prototypes to be further evaluated and refined by experts.

One caveat of our results is that the optimized model does not perform as well on data that was not used for the optimization. In general the predicted patterns of expression for the evaluation time courses (see, for example, Figures 8 and S3) are not of high enough quality to draw conclusions about the state of the system under different conditions given only the simulated data. Because of this uncertainty, the ability to estimate confidence for predicted behavior in the absence of observed data (such as was available here) is limited at best. However, the performance of the resulting optimized model on the data sets obtained from the other conditions indicates that it can make predictions with a statistically significant performance overall and performs very well for specific clusters. Universally the models optimized on each treatment had the least cross-predictive capability on the brief ischemia preconditioning time course. Examination of the gene regulation within this dataset reveals that, as a whole, the dynamic expression is dramatically different than the other three conditions (LPS, CpG-ODN, saline; see Table 2). Thus, since preconditioning with brief ischemia uniquely affects many of the dynamic changes in gene expression, we would expect that the model would not perform as well on this dataset. This result suggests that our model construction process may be most applicable for conditions that are similar in functional nature to those used for the optimization, such as the Toll-like receptor ligands LPS and CpG-ODN. We are currently working on ways to optimize the model to the entire set of preconditioning treatment time courses, while at the same time preserving the generalizability of the final model. Our current work provides a model with potential for applications that could directly impact development of new and/or improved treatments that result in preconditioning against stroke.

We chose to use correlation to compare the predicted expression patterns with observed patterns. Previously, the root mean square deviation (RMSD) measure or other similar error measures have been used to evaluate performance for similar problems. Unlike measures of error, correlation does not account for similarity in the magnitude of the two vectors being compared. Rather, it simply compares the relative patterns of expression. For this study we were more interested in getting the overall pattern of expression correct rather than matching absolute magnitude, which corresponds to fold-change in expression relative to baseline in this case. Models optimized using RMSD as a fitness criteria reached a mean normalized RMSD (error as percentage of the expression range) of approximately 30%, which is not exceptional. Evaluating these RMSD-optimized models using correlation revealed that they had essentially the same performance as the un-optimized model and many clusters showed trivial behavior, monotonically increasing or decreasing (data not shown). For some practical applications of these models a correlation-based approach would be insufficient. For example, when predicting expression levels that are associated with a phenotype that manifests as differences in magnitude between outputs under different conditions, an error measure would need to be used, potentially combined with correlation to ensure that patterns of expression are accurate.

An important limitation of the current model is that the experiments all include a significant disruption of the system in the middle of the time course in the form of the ischemic stroke induced at hour 72. Our dynamic model does not explicitly incorporate this event, which dramatically alters the regulation of gene expression. The effect of the stroke on gene expression is captured in this experiment, but is only poorly understood. Therefore, using the current dataset including stroke as an input to the model is not possible. An implicit assumption in our modeling approach is that the relationships between regulators and targets are fixed. The Inferelator approach and our dynamic ODE optimization process both aim to define these relationships based on existing data. The optimization process therefore learns the stroke stimulus from the data. We can predict the effects of changing early expression of particular clusters on the eventual output (ischemic injury) given our current model, but further experiments would be needed to validate these predictions. An important next step for model construction will be to consider the disruption in the model explicitly.

The time course experiments used in this analysis all had five time points. This is a limited number of points to parameterize the relationships between 25 clusters. Combining networks to create injurious and protected networks alleviates this limitation to a certain extent in terms of confident regulatory relationships, but the resulting models should be considered to be underdetermined. Including more data points in these models will be necessary to strengthen these models. Finally, a significant limitation of our models is that the clusters used as functional modules are still quite large and are likely to perform multiple functions. This fact prevents inference of mechanistic relationships between regulators and functional processes and pathways that would be desirable for this kind of approach. However, our validation results indicate that the functional modules defined are fairly coherent and supported by external data sources. We believe that the inclusion of more data points, which would allow parameterization of larger models, will allow the use of smaller, more focused clusters in the model. Additionally, development of methods to better delineate functionally coherent modules [23], [26] will allow the models to be more biologically interpretable. Further analysis will be required to determine if successful prediction for these clusters is biologically relevant.

The specific successes and failures of the dynamic model may provide important information on the role of certain gene clusters in neuroprotection and stroke. For example, the model shows strong predictive value for gene expression in clusters 14 and 15 for LPS, CpG-ODN, and brief ischemic preconditioning but has low predictive values for saline and sham (Table 2; Figure S3). Preconditioning with LPS, CpG-ODN, or brief ischemia all provide protection against the cerebral ischemic event in this model while the saline treatment leads to significantly worse damage. Evidence strongly indicates that these preconditioning paradigms alter the gene regulation that occurs prior to and following stroke to promote neuroprotection [2], [3], [18]. Thus, the clusters that are most predictive for all three preconditioning paradigms may reveal gene regulation that is important for neuroprotection. Clusters 14 and 15 functionally represent genes related to inflammation and apoptosis. Cluster 14 contains the gene tumor necrosis factor alpha (TNFα), which is a gene that is critical to preconditioning-induced protection. This is demonstrated by the loss of the neuroprotective effects of LPS and CpG-ODN preconditioning in mice deficient in TNFα [18], [33], [34]. Additionally, cluster 15 contains many interferon (IFN)-stimulated genes. A recent study that utilized the genomic brain data that corresponded to the genomic blood dataset utilized in this study showed that LPS, CpG-ODN, and brief ischemic preconditioning commonly reprogrammed the response to ischemia towards IFN regulated gene expression [1]. This suggests a role for IFN signaling in neuroprotection since the IFN-associated genes were only present in the preconditioning-induced neuroprotective environment. Consistently, the importance of IFN signaling is highlighted by the loss of preconditioning-induced protection against cerebral ischemia in IFN-regulatory factor 3 and 7 deficient mice [1], [35]. Finally, the dynamic model showed strong predictive ability in cluster 2 for LPS and CpG-ODN, but not for the other conditions. Cluster 2 is functionally representative of general metabolic processes. Research investigating the genomic response following stroke in mice preconditioned with brief ischemia shows that brief ischemia dramatically alters the expression of genes that are important to metabolism [2]. This shift in metabolic gene regulation may explain why the dynamic model did not accurately predict the outcome for brief ischemic preconditioning in cluster 2. Taken together, the results of the dynamic model appear to reflect some of the biological commonalities and discrepancies between the datasets. While the goal is to create a dynamic model that is predictive of multiple conditions in stroke, the results of this dynamic model already demonstrate its usefulness in understanding and discriminating which functional genes clusters are potentially important to each preconditioning paradigm, neuroprotection, and stroke.

Comparison of models derived from the neuroprotective and injurious states revealed that a number of predicted edges seemed to be different, either in presence and absence of an edge or in the sign of the weight of the edge (Figure 8). Examination of these differences shows that all the outgoing edges from cluster 5 are different in the neuroprotective versus injurious models. This suggests that cluster 5 may play a particularly pivotal role in neuroprotection, either in establishment of neuroprotection or post-stroke response to injury, or both. There are several members of this cluster that play known roles in endotoxin tolerance, a process thought to be closely related to neuroprotection against stroke [1]. Three negative regulators of signaling, Dusp1 (Mkp-1), Irak3 (IrakM), and Socs1 are members of the cluster, and knock-out mice have been shown to be more susceptible to endotoxin shock. Additionally, NFkβiα and NFkβiz, both inhibitors of NFkβ activity, are present in this cluster. We and others have shown that NFkβ activity is associated with protection against stroke [36]. In the current analysis no definitive links can be drawn between individual genes and importance in neuroprotection, but these observations are consistent with the idea that negative regulation of TLR pathways and NFkβ is pivotal in the process of neuroprotection [37]. Future modeling efforts will include specific investigation of the role of TLR inhibition in neuroprotection.

We have shown how transcriptomic data can be used to construct a dynamic model of gene expression at the level of functional modules in a largely automated way without the use of any prior knowledge about the system. The model is capable of accurately predicting the expression levels of component modules given only an initial starting state for key functional clusters. Our study also identifies several caveats and limitations that remain to be addressed, either through addition of more detailed expression data to our existing process, or by refinement of our methods. To our knowledge this is the first application of a modeling approach like this to data from a complex disease process in a vertebrate organism, specifically a mouse model of ischemia. Our approach provides a way to formulate prototype dynamic models from high-throughput data that can provide valuable insight into disease processes.

## Methods

### Data sets and processing

Microarray data were obtained from a transcriptional study of a mouse model of neuroprotection during stroke [1]. The dataset used for modeling is the accompanying blood samples from the previously published brain transcriptional analysis [1]. In brief, groups of C57BL/6 mice (n = 4/treatment/time) received either preconditioning alone, preconditioning plus injurious ischemia (45 min MCAO), or injurious ischemia alone. Preconditioning paradigms included: LPS (0.2 mg/kg; i.p.), CpG (0.8 mg/kg; i.p.), saline (i.p.), short-term MCAO (12 min), or sham MCAO (12 min). For groups receiving preconditioning alone, mice were euthanized at 3, 24 or 72 hr post preconditioning. In groups receiving preconditioning plus injurious ischemia, MCAO was performed 72 hr following the preconditioning stimulus and mice were euthanized at either 3 or 24 hr post occlusion. Six untreated mice were included as a baseline control group. Microarray assays were performed in the Affymetrix Microarray Core of the Oregon Health & Science University Gene Microarray Shared Resource. Labeled cRNA target was quality-checked based on yield and size distribution. Quality-tested samples were hybridized to the MOE430 2.0 array. The array image was processed with Affymetrix GeneChip Operating Software (GCOS). The original. CEL files have been deposited in the Gene Expression Omnibus under the accession number GSE32529. We used the robust multichip average method (RMA; [38]) normalized probe intensities to evaluate significantly changing probe sets and filtered for p-value<0.05 and fold changes greater than 2.0 to give 7352 significant probe sets.

### Clustering, functional enrichment and modularity

Hierarchical clustering was used to define functional modules from the filtered transcriptional data using the hclust command in the R statistical software (http://www.r-project.org/) and Ward's linkage [39]. The resulting hierarchical tree was used to divide the data into 5 to 50 clusters (in steps of 5). Each set of clusters was then assessed for functional modularity using the following approach. Clusters were assessed for functional enrichment using the GOStats library in R [40]. Each cluster was used as a set of genes to determine functional enrichment versus all other differentially expressed genes.

A functional coherence score was calculated by counting the number of genes appearing in at least one functional category that was enriched at a given threshold of significance over all clusters in that set. The pseudocode for the algorithm is as follows:

 Use hierarchical clustering to construct a tree of gene expression profiles

 For each clustering division:

  For each cluster present at this division:

   Calculate functional enrichment in this cluster versus all other genes

   Identify functional labels that are significantly enriched in the cluster versus all other genes at a specified level of significance

   Count number of genes that are annotated with at least one significant label (Gc)

  Functional coherence for this division is the sum of Gc over all clusters in that division

This metric provides a measure of functional modularity that is easy to interpret and can be adjusted by varying the significance threshold.

### Inferelator model inference

The Inferelator [13], [14] version 1.1 was used to infer a predicted causative gene regulation model. In the inference the mean expression pattern of each cluster was used both as a target for inference (i.e. to predict expression) and as a potential regulatory influence. Although the Inferelator allows use of non-linear combinations of regulatory influences in the inference process, we did not consider these in our model to allow the treatment of the resulting model as a linear system of coupled ODEs. The performance of the model was evaluated by constructing five independent models, each leaving one time course out for evaluation as previously described [23]. The performance results were calculated as the mean of performance results for each cluster, normalized by the number of genes in that cluster [23]. This initial model requires that the expression levels of the input regulatory influences, in this case other clusters, are known and that these can be combined to provide a predicted expression pattern for the target cluster.

To derive an initial model for dynamic simulations the coefficients for each regulatory influence were averaged over each of the five evaluation models, and those influences that were inferred in fewer than three models were excluded. Regulatory influences with coefficients having an absolute value less than 0.1 were also excluded. This eliminates regulatory influences with low impact on the final model and limits the final number of regulatory influences in the model.

### Dynamic simulations

Our main model for the time evolution of gene cluster expression can be described as follows. Suppose we have *N_c_* clusters, and let *c_i_* represent the mean expression level of cluster *i* for 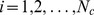. Our model equations are:
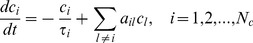
(1)where *τ_i_* is a cluster expression decay constant and 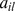 is the sum of weights for the influence of all regulators in cluster *l* on cluster *i*. Note that the right-hand side can be written as a single sum 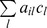 if we define 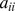 to be −1/*τ_i_*. The system of ODEs may be written in matrix-vector form as:
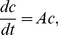
(2)where 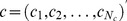 and
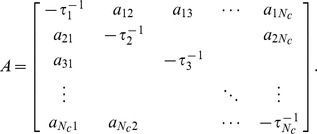
The solution is readily calculated at any time given the matrix *A* and initial expression levels.

Initial values for the system were taken to be the expression value of each cluster at *t_0_* = 3 h, the earliest time point, for each time course. Performance of the simulation was evaluated as the mean of the correlations of the simulated expression for each cluster with the observed expression for that cluster at times 3, 24, 72, 75, and 96 h post-treatment, normalized by the number of genes in that cluster (analogous to the performance metric used for the Inferelator-based model evaluation). We provide our R code for these simulations as Dataset S1.

### Optimization

To optimize the initial matrix for dynamic simulation over time, we employed a standard simulated annealing approach that was separated into two stages. In the first stage 25 independent simulated annealing runs of 5000 steps each were performed using the initial matrix *A* derived using Inferelator 1.1 as a starting point. The initial probability of accepting a deleterious change was set to 0.80. The cooling factor was 0.002 per step and the size of perturbations in the matrix elements was determined on a random relative scale:
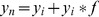
(4)where *y_n_* is the new perturbed value in the matrix, *y_i_* is the initial value for that location and ***f*** is a pseudorandom uniform deviate between −0.6 and 0.6. At each step the probability of adding an influence in a location that had previously been 0 was set to 0.1%, and the probability of removing a non-zero influence was set to 1%. Aside from these exceptions perturbations were only allowed for non-zero values in the matrix. This first stage provided a set of optimized models with high heterogeneity to explore the variable space around the initial matrix. The matrix producing the best performance in the first stage was chosen to serve as the initial matrix for the second stage of optimization.

The second stage of optimization was performed in the same manner as the first to generate 25 additional models, but was allowed to proceed for 25000 steps. The initial probability of accepting deleterious perturbations was set to 10%, with all other parameters remaining the same as in the first stage. This stage allowed the simulation to explore the variable space around the best performing matrix from the first stage in order to refine the model. The best performing matrix from this stage was chosen as the final model. A third stage of optimization was found to provide no improvement to the best models and is not included in the results (data not shown).

For simulated annealing optimization the fitness function was the performance of the simulation, defined as the correlation between the simulated and observed expression levels, for each pretreatment time course. Initial values for the simulation were taken as the 3 h time point from the corresponding time course. The final model was then evaluated for its performance on the other time courses using the 3 h time point from the other time courses as initial model values.

To evaluate the significance of the results obtained by optimization we compared performance of the optimized model to the model using randomly perturbed matrices, as was done with the initial matrix prior to optimization. To study the dependence of the optimized model on the initial matrix, the matrix was perturbed randomly and the simulated annealing process described above was repeated on the perturbed matrices. To assess the dependence of performance on the initial Inferelator-derived values for the matrix, non-zero values in the matrix were randomly permuted (referred to here as resampled) such that the structure of the model was preserved. This was repeated 100 times and a p-value calculated based on the distribution of performance values from the resampled matrices. A second test in which random numbers drawn from a uniform distribution between −2 and 2 were assigned to non-zero values in the matrix (uniform) was also used to assess the impact of the initial values. Finally, the dependence of performance on the structure of the model was assessed by randomly permuting all values in the matrix (scramble). These approaches were also used to evaluate the significance of the performance of the final optimized matrix.

### Validation of model using external datasets

To validate the cluster-to-cluster relationships in our model we used edges from four different sources. Regulatory interactions (regulator to target relationships) derived from CHIP experiments were obtained from the ChEA database [27] giving 2506 edges between 1587 genes included in our model. Protein-protein interactions were obtained from the Human Proteome Research Database (HPRD) [28], and identifiers were mapped to mouse using gene symbols giving 2974 edges between 1393 genes included in our model. Though some interactions identified in human may not be preserved in mouse, overall they are likely to be consistent across organisms [41]. Known gene regulatory interactions were obtained from the Molecular Signatures Database (MSigDB) [29] giving 1895 edges between 570 genes in our model. Functional interactions derived from computational integration of multiple data source were obtained from high-confidence (score>0.5) interactions made by MouseNet [30] giving 2307 edges between 1023 genes in our model.

We counted the number of interactions between clusters in our model then compared that number to counts from analyses with randomly rewired edges (same genes and number of edges with randomized gene labels), repeated 1000 times to obtain p-values for the count. For undirected edges (HPRD and MouseNet) relationships were counted for both directions. For relationships present in our model (optimized weight between two clusters is non-zero) we counted a true positive (TP) if the p-value was less than 0.05 for the interaction count, otherwise counted a false negative (FN). Likewise, for relationships not present in our model a true negative (TN) was counted if the p-value was more than 0.05, otherwise a false positive was counted. Accuracy was calculated as TP+TN/(TP+TN+FP+FN).

## Supporting Information

Dataset S1 **Prototype code for dynamic simulations performed in the paper.**
(TGZ)

Figure S1 **Distribution of edge counts in LPS optimized model ensemble.** The number of times an edge appears in the 25 models from the LPS-optimized ensemble is shown as a histogram.
(PDF)

Figure S2 **Edge consistency in model ensemble.** Clusters containing more than 5 genes are shown (green squares) as determined by our functional clustering approach. The influences between clusters are shown as directed edges with arrows indicating a positive influence (activation) and T lines indicating a negative influence (repression). Line coloring indicates the number of models in the LPS-optimized ensemble that the edge appears in, grey edges are not present in the LPS-optimized model but were present in the original model.
(PDF)

Figure S3 **Cross-prediction plots for the LPS-optimized model.** Relative expression levels (log_2_ fold change expression) are plotted over time (X axis) for the predicted (green line) and observed (black line) expression levels for the indicated cluster.
(PDF)

Table S1 **Optimization results for model ensembles from all pretreatments (data file).**
(XLSX)

Table S2 **External validation of network model using different interaction sets.**
(XLSX)

Table S3 **Edge consistency between models and injurious versus non-injurious combined models (data file).**
(XLSX)
