# Supplementary figures and images for: Modeling Dynamic Regulatory Processes in Stroke
Source: PLoS Comput Biol. 2012 Oct 11;8(10):e1002722. doi: 10.1371/journal.pcbi.1002722 (PMC3469412; doi:10.1371/journal.pcbi.1002722)

Figure S1

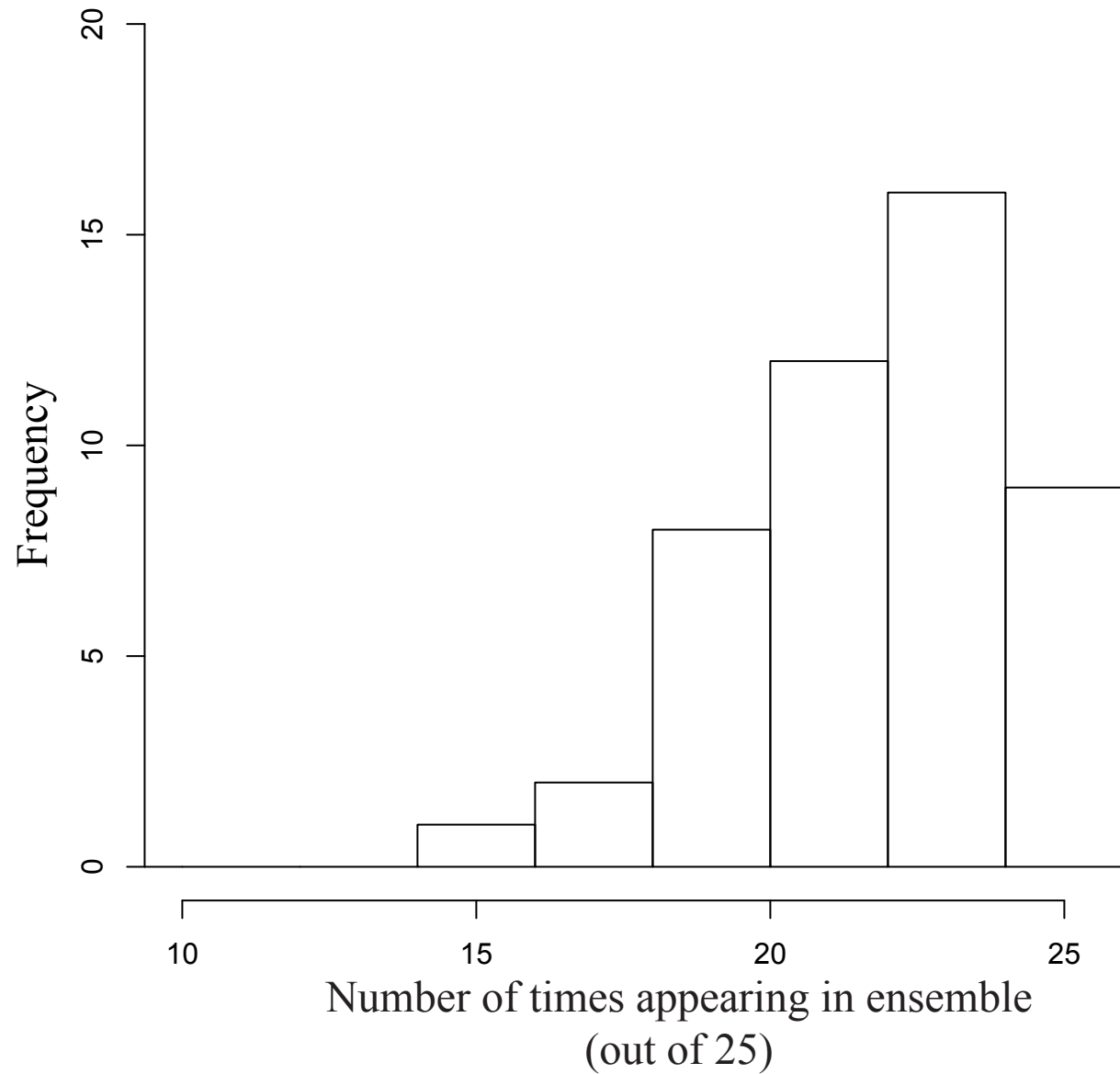

Supplement: Figure S1 — Distribution of edge counts in LPS optimized model ensemble. The number of times an edge appears in the 25 models from the LPS-optimized ensemble is shown as a histogram. (PDF) [file pcbi.1002722.s002.pdf]

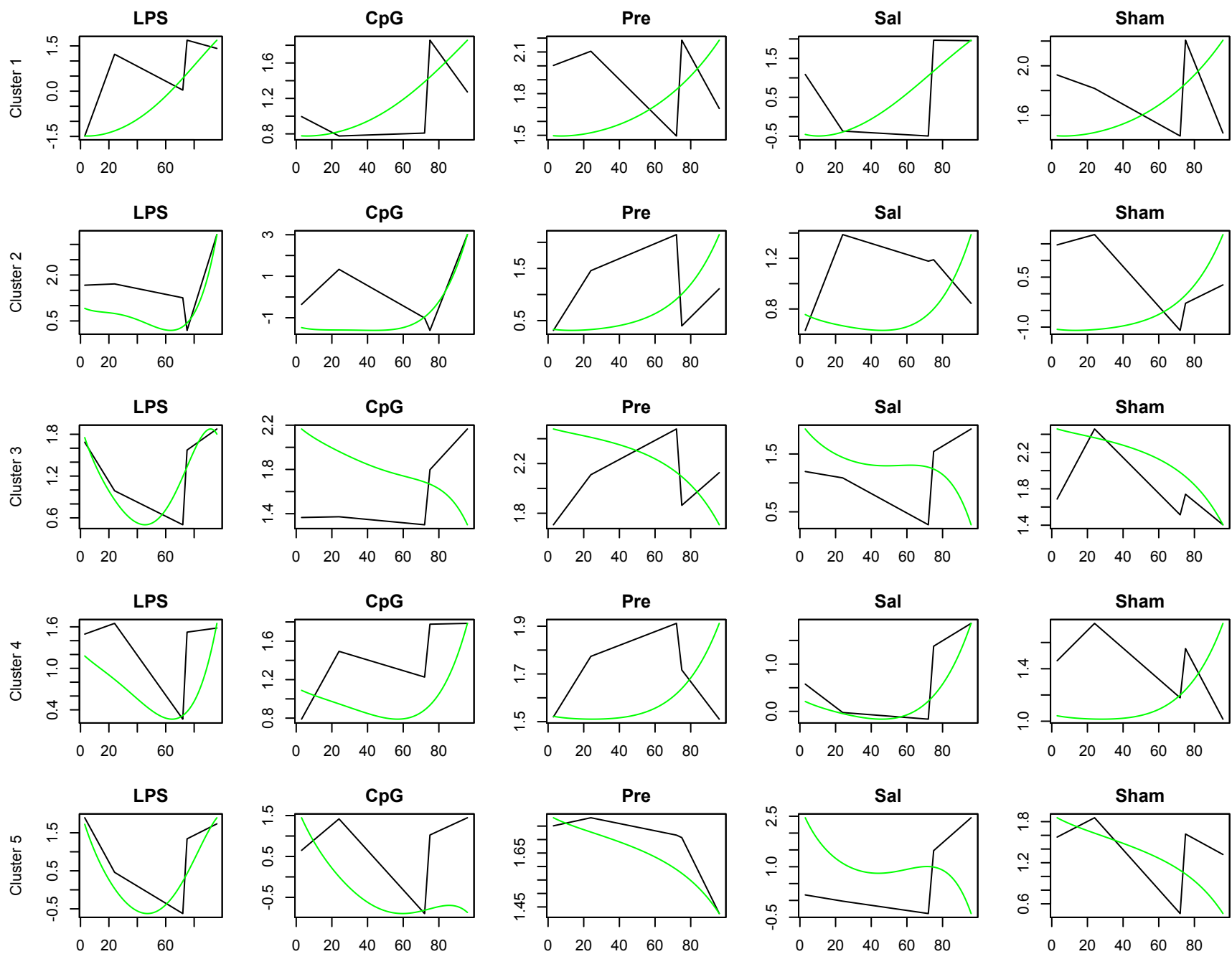

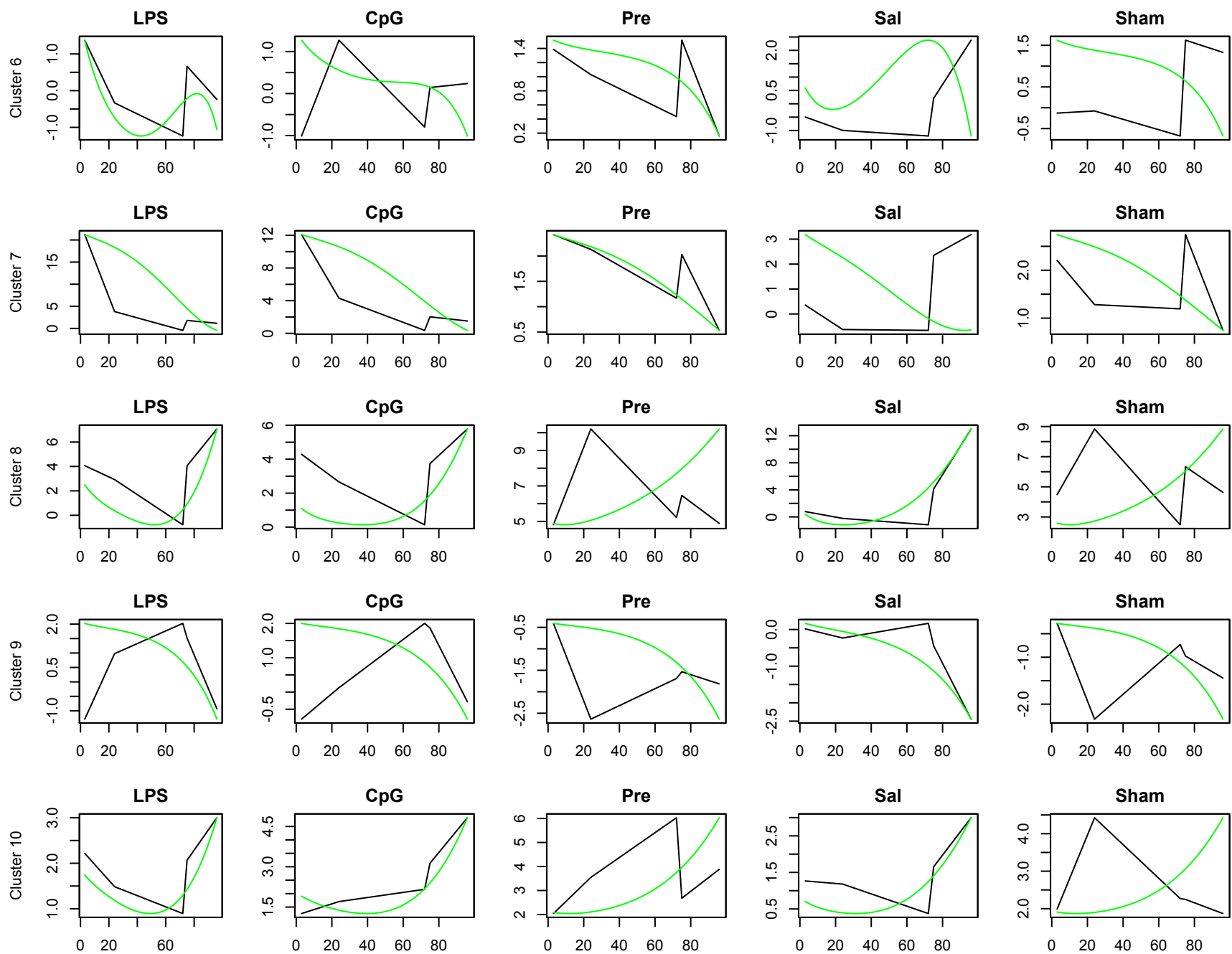

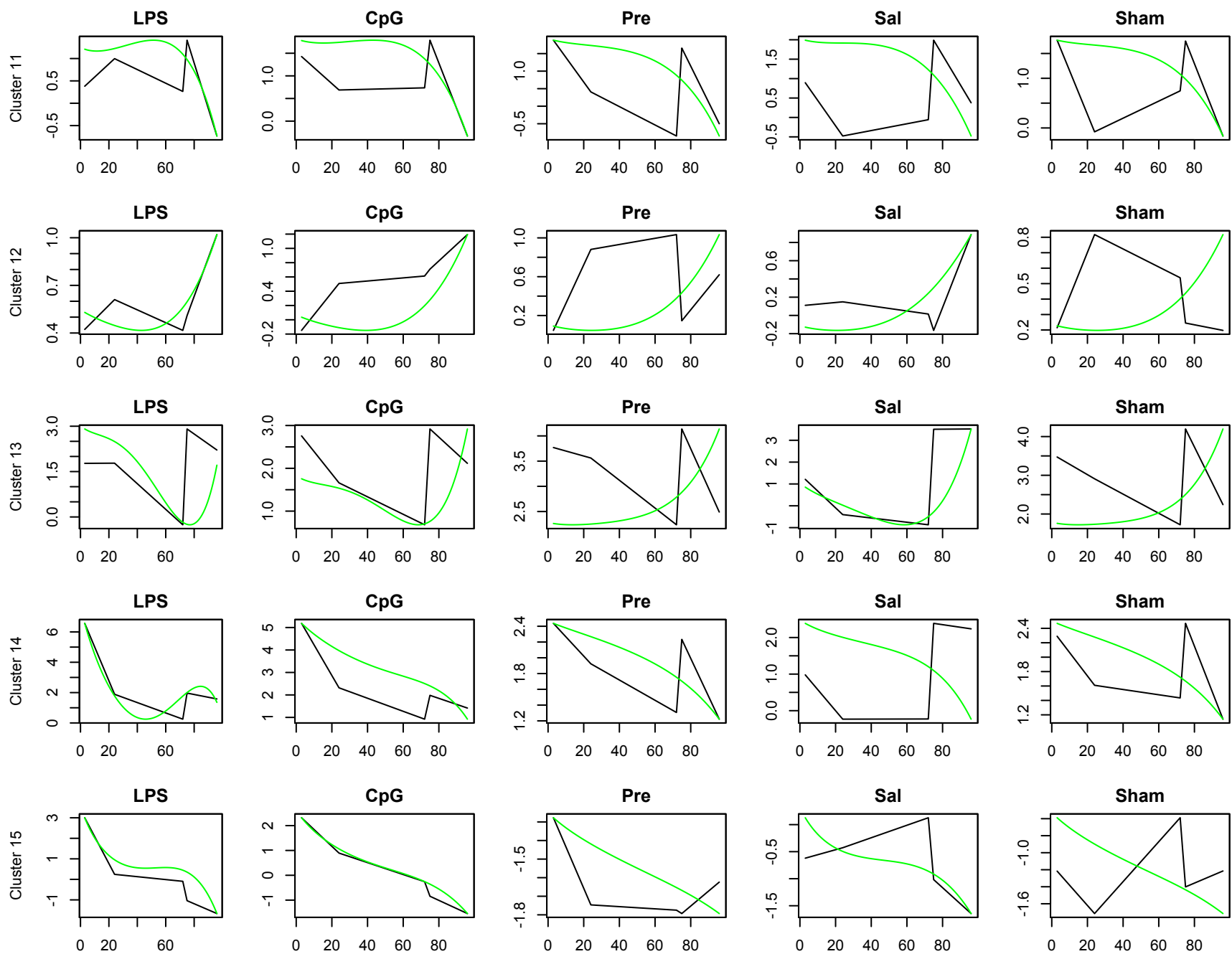

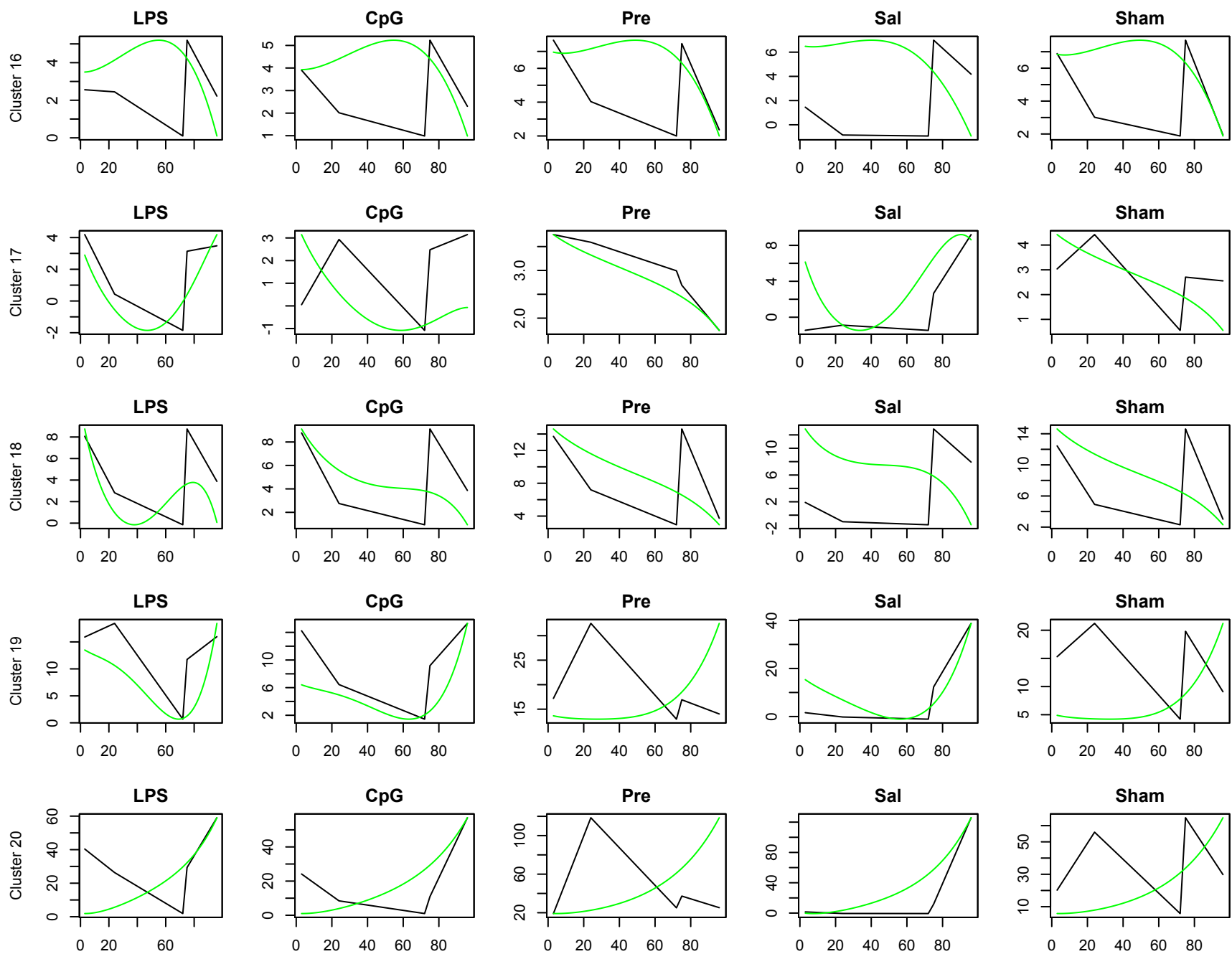

Supplement: Figure S3 — Cross-prediction plots for the LPS-optimized model. Relative expression levels (log2 fold change expression) are plotted over time (X axis) for the predicted (green line) and observed (black line) expression levels for the indicated cluster. (PDF) [file pcbi.1002722.s004.pdf]
