# Supplementary material for: Modeling Dynamic Regulatory Processes in Stroke
Source: PLoS Comput Biol. 2012 Oct 11;8(10):e1002722. doi: 10.1371/journal.pcbi.1002722 (PMC3469412; doi:10.1371/journal.pcbi.1002722)
Supplement: Figure S2 — Edge consistency in model ensemble. Clusters containing more than 5 genes are shown (green squares) as determined by our functional clustering approach. The influences between clusters are shown as directed edges with arrows indicating a positive influence (activation) and T lines indicating a negative influence (repression). Line coloring indicates the number of models in the LPS-optimized ensemble that the edge appears in, grey edges are not present in the LPS-optimized model but were present in the original model. (PDF) [file pcbi.1002722.s003.pdf]

Figure S2

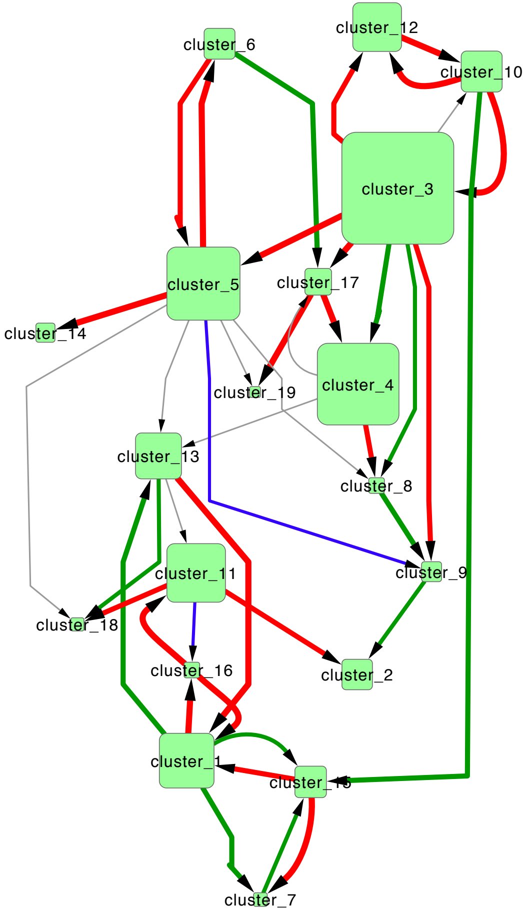

Not represented in LPS-optimized model

Represented in fewer than 20 models

Represented in 20-22 models

Represented in 23-25 models
